# Iron metabolism mediates the relationship between Vitamin C and hepatic steatosis and fibrosis in NAFLD

**DOI:** 10.3389/fnut.2022.952056

**Published:** 2022-09-08

**Authors:** Zhengyu Hu, Yan Li, Bingwei Ma, Saifei Lei, Xingchun Wang

**Affiliations:** ^1^Department of General Surgery, Shanghai Tenth People's Hospital, Affiliated to Tongji University School of Medicine, Shanghai, China; ^2^Department of Gastroenterology, Shanghai Tenth People's Hospital, Affiliated to Tongji University School of Medicine, Shanghai, China; ^3^Center for Pharmacogenetics, Department of Pharmaceutical Sciences, University of Pittsburgh, Pittsburgh, PA, United States; ^4^Department of Endocrinology and Metabolism, Shanghai Tenth People's Hospital, Tongji University, School of Medicine, Shanghai, China

**Keywords:** NAFLD, Vitamin C, ferritin, hepatic steatosis, hepatic fibrosis

## Abstract

Vitamin C (Vit C) and iron metabolism are closely related to metabolic disorders. However, the relation between iron storage protein ferritin and Vit C has not been elucidated. We aimed to investigate the crosstalk between Vit C and ferritin and its implications on non-alcoholic fatty liver disease (NAFLD). Clinical information of 3,614 subjects was obtained from the NHANES Public Data 2017–2018. FibroScan data, which estimates liver steatosis and fibrosis and Vit C, were selected to assess factors influencing NAFLD in this cross-sectional study. Ferritin and Vit C among different categories of liver steatosis and fibrosis were assessed by CAP and E value. Logistic regression and RCS models were used to analyze the correlations. *In vitro* study in hepG2 were conducted to validate the regulations. Ferritin increased while Vit C decreased with more severe hepatic steatosis and hepatic fibrosis (all *P* < 0.001). Logistic regression models indicated that increased serum ferritin was a risk factor for NAFLD while increased Vit C was a protective factor for NAFLD and hepatic fibrosis after adjusting the continuous and categorical variables. Vitamin C was negatively associated with ferritin. Further mediation analysis identified that ferritin mediates the impact of Vit C on NAFLD (*P* < 0.05) and cirrhosis (*P* < 0.001). The experiments on cellular level suggested Vit C alleviated PA/OA induced steatosis and maintains iron homeostasis through inhibiting PA/OA induced upregulation of iron bound protein ferritin and labile iron pool (LIP) induction in hepG2 cells. In conclusion, Vit C was a protective factor, whereas ferritin was a risk factor for hepatic steatosis and fibrosis. Vitamin C alleviated NAFLD and maintained iron homeostasis via ferritin suppression and LIP induction.

## Introduction

The growing prevalence of non-alcoholic fatty liver disease (NAFLD) is a major health problem worldwide (1). More attention is being paid to NAFLD and its association with obesity, type 2 diabetes mellitus (T2DM), and metabolic syndrome (MetS) (1). Non-alcoholic fatty liver disease may also progress to non-alcoholic steatohepatitis (NASH), hepatic fibrosis, and even hepatoma carcinoma (2). However, the underlying mechanisms of the onset and progression of NAFLD are not yet fully understood.

Excessive fat deposits in the liver may lead to the loss of vitamin homeostasis (3). Vitamin C (Vit C) deficiency occurs in NAFLD as well as NASH (4). Cross-sectional studies of a large cohort of 789 subjects and 3,471 subjects showed that Vit C intake may be protective against NAFLD (5, 6). A randomized controlled trial (RCT) that included 84 patients with NAFLD showed that 12 weeks of Vit C supplementation improved liver health (7). As a water-soluble vitamin, Vit C acts as an enzymatic co-factor and reducing agent, and is required for many biological processes, such as collagen synthesis and β-oxidation. Further, Vit C inhibits liver steatosis by reducing oxidative stress (8, 9). Despite the increasing beneficial evidence of Vit C against NAFLD, little is known about the underlying mechanisms of Vit C intervention.

Iron overload has effects on metabolic diseases such as obesity, T2DM, and NAFLD, which are associated with glucose-lipid metabolism (10). As an essential iron storage protein in cells, ferritin plays a critical role in cellular biological functions, metabolism, and iron homeostasis (11). As a measure of iron loading, serum ferritin in an obese adult is correlated with adipose tissue dysfunction and liver fat (12). A cross-sectional study showed that serum ferritin levels were significantly higher in subjects with NAFLD, and NAFLD was a determinant of serum ferritin levels (13). Ferritin may be a potential non-invasive predictive biomarker for NAFLD, but the mechanisms with NAFLD have not been fully elucidated (14). However, data linking hepatic iron with NAFLD remains vague, and with conflicting evidence (15).

Given this premise, we aimed to investigate the association of Vit C with ferritin and the crosstalk in NAFLD. In our study, we analyzed the changes in serum Vit C and ferritin in NAFLD and examined their association to clarify the underlying mechanism.

## Materials and methods

### Data source

The National Health and Nutritional Examination Survey (NHANES) is a cross-sectional nationwide program aimed at monitoring the physical condition and nutritional status of the US population conducted by the National Center for Health Statistics (NCHS) at the Centers for Disease Control and Prevention (CDC) (Available online: https://wwwn.cdc.gov/nchs/nhanes/Default.aspx), as described in our published study (16). The data in our study include socio-demographic issues, dietary schemes, and medical data, which were obtained through interviews, professional physical examinations, and laboratory measurements. The program was approved by the Institutional Review Board (IRB) of the NCHS, and all participants or participants' agents signed informed consent forms.

### Patients

We collected data from 9,254 participants from the NHANES Public Data 2017–2018. Subjects with missing information on iron metabolism and Vit C (36.8%) were excluded. Partially completed and ineligible liver ultrasound transient elastography (LUTE) samples were also excluded. Other exclusion criteria were as follows: (1) those with HBV antigen or HCV RNA positivity and (2) participants who were under the influence of alcohol. Overall, 3,614 eligible subjects were included in the analysis. The data details of patients were shown in Supplementary Figure 1.

### Diagnostic criteria and grading standard

During the 2017–2018 NHANES cycle, liver transient elastography was performed to assess the degree of liver steatosis and sclerosis. All NHANES health technicians and examiners completed a 2-day training program using FibroScan^®^ and passed the exam. MEC staff have been regularly supervised by investigators and medical epidemiologists specializing in chronic liver disease, thus ensuring the quality of LUTE.

Based on a recent study done by Eddowes et al. median CAP values ≥274, 290, and 302 dB/m were considered to be indicative of S1, S2, and S3 steatosis, respectively (17). Median stiffness values ≥8.2, ≥9.7, and ≥13.6 kPa were considered markers of significant fibrosis (≥F2), advanced fibrosis (F3), and cirrhosis (F4), respectively (17).

To create a real fit model as possible, potential confounders were also included as covariates in our study, which include age, sex (male or female), race (non-Hispanic white, non-Hispanic black, Mexican American, Hispanic, and other races), education level (below high school, high school or equivalent, college, or above), marital status (married and single), and household poverty-to-income ratio (PIR) ( ≤ 1. 30, 1.31–3.5, >3.5, and others), BMI (<25 and ≥25 kg/m^2^), strenuous recreational activity (yes or no), moderate recreational activity (yes or no), smoking status, history of hypertension, diabetes, and chronic kidney disease. Smoke exposure (active or passive smoking) status was determined by serum cotinine concentration with a cut-off value of 14 ng/ml. The diagnoses of hypertension, diabetes mellitus, and chronic renal failure in the study subjects either met the clinical criteria or were based on the medical history (history of relevant pharmaceutical use) from the questionnaire. Participants' dietary data were sourced from the What We Eat in America (WWEIA) project. The average of two 24-h nutrient intakes was used to estimate the daily nutrient intake of participants in the past month.

Information regarding diabetes, hypertension, and chronic renal failure was based on self-report questionnaires. Hypertension was defined as systolic blood pressure (SBP) >140 mmHg or diastolic blood pressure (DBP) >90 mmHg (18). Diabetes was diagnosed as fasting plasma glucose (FPG) levels ≥7.0 mmol/l or 2-h postload blood glucose (PBG) ≥11.1 mmol/l (19, 20). Dyslipidemia was defined as fasting plasma triglyceride (TG) ≥1.7 mmol/l, fasting high-density lipoprotein cholesterol (HDL-C) <1.04 mmol/l, low-density lipoprotein cholesterol (LDL-C) ≥3.37 mmol/l, or total cholesterol (TCH) ≥5.18 mmol/l (21). Hyperuricemia was defined as serum uric acid (UA) ≥7 mg/dl (≥417 μmol/L) in men and ≥6 mg/dl (≥357 μmol/L) in women (22). Additionally, MetS was defined by three out of four components as follows (23): (1) overweight and(or) obesity [body mass index (BMI) ≥25 kg/m^2^], (2) FPG ≥6.1 mmol/l and(or) PBG ≥7.8 mmol/l and(or) diagnosed diabetes, (3) hypertension: BP ≥140/90 mmHg or positive history of hypertension or on any hypertensive treatment, (4) dyslipidemia.

### Cell cultures and establish of steatosis models and treatment

HepG2 cell lines were obtained from Shanghai Tenth People's Hospital of Tongji University and cultured at 37°C and 5% CO_2_ using DMEM-HG (Gibco, USA) containing 10% fetal bovine serum (Gibco, USA) and 1% v/v penicillin–streptomycin. Oleic acid (OA) (A502071, Sangon Biotech, Shanghai, China) and palmitic acid (PA) (A600497, Sangon Biotech, Shanghai, China) were dissolved in 5% BSA. Cellular steatosis was induced by the addition of 600 μM OA and 300 μM PA. Intracellular lipids were verified with the Oil Red O staining kit (G1262, Solarbio, Beijing, China). Cells were treated with different concentrations of Ascorbic acid (Vitamin C, A92902, Sigma-Aldrich, USA) for 24 h and harvested for subsequent experiments to test the effect of Vit C.

### CCK8 assay

HepG2 cells (5 × 10^3^/well) were plated into 96-well plates. Cells were then treated with fatty acids and different concentrations of Vit C for 24 h. Cell viability was measured using a CCK8 reagent (No. C0005, Topscience, Shanghai, China) according to the manufacturer's instructions.

### Measurement of glucose-lipid indexes

Intracellular TG and TCH concentrations were measured according to the instructions of the TG (F001-1-1, Nanjing Jiancheng Bioengineering Institute, Nanjing, China) and TCH assay kits (F002-1-1, Nanjing Jiancheng Bioengineering Institute, Nanjing, China), and the values were corrected using the protein concentration of the samples. The glucose concentration in the medium was measured according to the instructions of the glucose assay kits (A154-1-1, Nanjing Jiancheng Bioengineering Institute, Nanjing, China), and the values were corrected using the cell numbers of the samples. The cell numbers of the samples were tested by an automated cell counting chamber (SD100-002, Nexcelom Bioscience, Shanghai).

### Western blotting

Total proteins were extracted from cells using RIPA lysis buffer (PC101, Epizyme, Shanghai, China) with protease inhibitor cocktail (GRF101, Epizyme, Shanghai, China). Equal amounts of proteins were transferred to polyvinylidene fluoride membranes and imaged on an Odyssey scanner (LI-COR Biosciences, USA) after incubation with primary ferritin heavy chain (FTH) (T57085, Abmart), ferritin light chain (FTL) (T56955, Abmart), and β-tubulin (M30109, Abmart) and secondary antibodies (#7054, #7056, Cell Signaling Technology). The primary antibodies were diluted at 1:1,000 and secondary antibodies were diluted at 1:2,000.

### The labile iron pool measurement

The iron-sensitive fluorescent dye Calcein-AM (40719ES, Yeason, Shanghai, China) was used to measure intracellular labile iron pool (LIP) levels according to the previously described method (24, 25). Briefly, cells were stained in 6-well plates using 0.1 μM Calcein-AM at 37°C for 15 min and washed three times with PBS to remove excess dye, then cells were detached using 0.25% trypsin and resuspended in 500 μl 1X Binding Buffer. The intracellular fluorescence intensity was immediately measured by flow cytometry (BD Biosciences, λ_ex_ 488 nm; λ_em_ 530 nm). Following this the cells were incubated with 100 μM deferiprone (HY-B0568, MedChemExpress, USA) at 37°C for 40 min (Shake every 5 min to avoid cell clumping) fully bind the free iron in the cells and run the flow assay again. The mean fluorescence intensity (MFI) was calculated by FlowJo software (v10.4) for each population of at least 10,000 cells. The difference in MFI (Δ*F*) after and before deferiprone treatment was used to reflect the level of LIP.

### Statistical analysis

Continuous variables are presented as the mean ± standard deviation (X ± SD). Categorical variables are presented as numbers (percent). All data analyses were performed using NANS-recommended weighting correction. Normally distributed data were compared with the independent sample *t*-test, while the non-normally distributed continuous data were compared using the Mann–Whitney U test. For comparison of categorical variables, the chi-square test was used. Pearson's correlation coefficient was used to analyze the correlations between the markers.

Restricted cubic spline (RCS) was performed to fit changes in odds of NAFLD across the Vit C and ferritin range. In addition, mediating effect analysis was used to investigate whether Vit C affects the development of NAFLD or liver fibrosis through serum ferritin mediation. We hypothesized that the dose-response relationship between Vit C (X) and NAFLD (Y) is mediated by serum ferritin (M). The total effect (TE) of Vit C was split into a direct effect (DE) and an indirect effect (IE) on NAFLD. According to Baron and Kenny et al. (26), an intermediate effect of M is considered to exist when the conditions of “statistically significant association between X and M” and “statistically significant association between M and Y” are met.

All statistical analyses were performed using R (version 4.0.2) and SPSS software (version 24.0) and involved the use of R packages such as “mediation,” “rms,” and graphical visualizations were generated by “ggplot2.” Statistical significance was set at *P* < 0.05.

## Results

### Ferritin and serum Vit C correlated to steatosis severity

Three thousand six hundred and fourteen eligible subjects were divided into four steatosis degree groups, S0 (normal, <274 dB/m), S1 (mild, 274–290 dB/m), S2 (moderate, 290–302 dB/m), and S3 (severe, ≥302 dB/m) according to the CAP values. The age (*P* < 0.001), the percentage of males (*P* < 0.001), and married populations (*P* = 0.002) tended to be higher in more severe hepatic steatosis. The obesity indicators: body weight, BMI, WC, and waist-to-hip ratio (WHR), liver injury markers: alanine transaminase (ALT), aspartate aminotransferase (AST), gamma glutamyl transpeptidase (GGT), and alkaline phosphatase (ALP) levels, glucose metabolic markers: FPG, fasting insulin (FINS), homeostasis model assessment of insulin resistance (HOMA-IR), and glycosylated hemoglobin (HbA1c), blood pressure related SBP, DBP, and comorbidities of metabolic disorders such as the rate of hypertension, diabetes, hyperlipidemia, hyperuricemia, and MetS increased with the increasing severity of hepatic steatosis (all *P* < 0.001). Lipid metabolic markers, TC, LDL-C, and TG increased while HDL-C decreased in the group with more severe hepatic steatosis (all *P* < 0.05). Additionally, factors related to the steatosis severity of NAFLD is complex. Interestingly, we found ferritin also increased significantly (*P* < 0.001) while serum Vit C decreased with more serve hepatic steatosis (*P* < 0.001) (Table 1).

**Table 1:** Characteristics of the study population according to CAP values^*^.

	**Categories of liver steatosis**				***P*-value**
	**S0**	**S1**	**S2**	**S3**	
Patients, *n* (%)	2,184 (60.7)	299 (8.4)	207 (5.2)	924 (25.7)	
**Demographics data**					
Gender					<0.001
Male	1,052 (48.9)	144 (53.5)	111 (51.0)	552 (61.7)	
Female	1,132 (51.1)	155 (46.5)	96 (49.0)	374 (38.3)	
Age (years)	41.6 ± 23.2	50.6 ± 20.2	51.6 ± 20.1	53.1 ± 18.5	<0.001
Ethnicity					0.249
Non-Hispanic White	706 (60.6)	88 (58.3)	70 (58.8)	345 (65.1)	
Non-Hispanic Black	543 (12.8)	68 (12.0)	38 (8.7)	151 (7.7)	
Mexican American	247 (7.4)	48 (10.7)	42 (15.5)	154 (10.3)	
Hispanics	201 (7.2)	20 (4.8)	16 (5.4)	85 (6.3)	
Asian	361 (7.1)	58 (8.9)	35 (8.6)	135 (6.3)	
Others	126 (4.8)	17 (5.4)	6 (2.9)	54 (4.4)	
Marital status					0.002
Married	784 (42.3)	154 (62.1)	115 (60.1)	524 (65.5)	
Non-married	1,400 (57.7)	145 (37.9)	92 (39.9)	400 (34.5)	
Education					0.007
Less than high school	309 (9.2)	56 (10.4)	38 (11.8)	173 (10.5)	
High school or equivalent	352 (19.9)	46 (17.4)	42 (25.8)	205 (27.1)	
College or AA degree	870 (48.5)	168 (67.2)	106 (56.3)	463 (57.2)	
College graduate or above	653 (22.3)	29 (5.1)	21 (6.1)	83 (5.2)	
PIR					0.950
≤ 1.30	573 (18.7)	69 (17.5)	46 (13.5)	239 (19.2)	
1.31–3.50	790 (32.7)	106 (29.2)	71 (32.5)	337 (35.6)	
>3.50	561 (38.9)	79 (42.8)	56 (43.5)	231 (34.2)	
Unknown	260 (9.7)	45 (10.6)	34 (10.5)	117 (11)	
**Physical examination**					
Smoke status					0.677
Yes	342 (15.6)	35 (11.7)	29 (14.0)	145 (15.6)	
No/Unknown	1,842 (84.4)	264 (88.3)	178 (86.0)	779 (84.4)	
SBP (mmHg)	120.1 ± 0.44	125.2 ± 1.13	125.5 ± 1.40	127.1 ± 0.62	<0.001
DBP (mmHg)	70.2 ± 0.24	73.3 ± 0.65	74.2 ± 0.85	76.2 ± 0.40	<0.001
Pulse (mmHg)	70.1 ± 0.29	68 ± 0.69	70.4 ± 0.88	70.7 ± 0.43	0.657
Height (cm)	165.1 ± 10.05	166 ± 10.18	166.1 ± 10.34	167.4 ± 10.22	<0.001
Weight (kg)	70.0 ± 17.98	83.8 ± 21.98	84.8 ± 20.15	94.7 ± 22.23	<0.001
BMI (kg/m^2^)					<0.001
Mean, SE	25.6 ± 5.85	30.3 ± 7.02	30.6 ± 5.95	33.7 ± 6.92	<0.001
<25	1,133 (51.8)	61 (20.3)	27 (13.0)	63 (6.9)	
≥25	1,051 (48.2)	238 (79.7)	180 (87.0)	861 (93.1)	
WC (cm)	88.8 ± 14.93	101.1 ± 13.91	103.4 ± 12.60	111.4 ± 15.13	<0.001
HC (cm)	99.2 ± 12.07	106.9 ± 12.83	107.9 ± 12.70	113.5 ± 14.39	<0.001
WHR	0.89 ± 0.08	0.95 ± 0.07	0.96 ± 0.07	0.98 ± 0.07	<0.001
Vigorous recreational activities					<0.001
Yes	453 (20.9)	51 (17.0)	35 (16.9)	135 (14.7)	
No/Unknown	1,731 (79.1)	248 (83.0)	172 (83.1)	789 (85.3)	
Moderate recreational activities					0.637
Yes	725 (33.4)	100 (33.3)	77 (37.2)	294 (31.8)	
No/Unknown	1,459 (66.6)	199 (66.7)	130 (62.8)	630 (68.2)	
**Comorbidity**					
Hypertension					<0.001
Yes	514 (23.5)	113 (37.7)	83 (40.1)	459 (49.7)	
No/Unknown	1,670 (76.5)	186 (62.3)	124 (59.9)	465 (50.3)	
Diabetes					<0.001
Yes	174 (7.9)	46 (15.3)	36 (17.4)	258 (27.8)	
No/Unknown	2,010 (92.1)	253 (84.7)	171 (82.6)	666 (72.2)	
Hyperlipemia					<0.001
Yes	1,007 (46.1)	194 (65)	141 (68.1)	722 (78.2)	
No/Unknown	1,177 (53.9)	105 (35)	66 (31.9)	202 (21.8)	
Hyperuricemia					<0.001
Yes	259 (11.9)	70 (23.7)	40 (19.3)	256 (27.6)	
No/Unknown	1,925 (88.1)	229 (76.3)	167 (80.7)	668 (72.4)	
MS					<0.001
Yes	354 (16.1)	99 (33.0)	77 (37.2)	500 (54.0)	
No/Unknown	1,830 (83.9)	200 (67.0)	130 (62.8)	424 (46.0)	
**Laboratory feature**					
Platelet count ( ×10^9^/L)	244.5 ± 63.68	246 ± 62.06	245.6 ± 67.76	244.9 ± 64.5	0.998
Albumin (g/dl)	4.14 ± 0.33	4.08 ± 0.32	4.06 ± 0.31	4.05 ± 0.32	<0.001
ALT (IU/L)	17.7 ± 13.97	20.9 ± 12.55	22.1 ± 12.12	27.6 ± 18.92	<0.001
AST (IU/L)	20.4 ± 10.17	20.4 ± 7.09	21.1 ± 8.28	23.7 ± 13.81	<0.001
GGT (IU/L)	21.8 ± 24.76	27.5 ± 25.38	31.4 ± 55.97	36.6 ± 39.34	<0.001
ALP (IU/L)	99.2 ± 67.22	87.7 ± 39.9	84.5 ± 38.26	88.5 ± 37.8	<0.001
FPG (mmol/L)	5.8 ± 1.34	6.3 ± 1.59	6.5 ± 2.04	7.2 ± 2.67	<0.001
FINS (mU/L)	10.0 ± 8.49	17.4 ± 42.13	16.4 ± 12.39	22.5 ± 26.96	<0.001
HOMA-IR	2.6 ± 2.34	4.6 ± 10.08	4.7 ± 3.72	7.7 ± 12.8	<0.001
HbA1c (%)	5.6 ± 0.78	5.8 ± 0.92	6.0 ± 1.20	6.3 ± 1.38	<0.001
LDH (IU/L)	160.3 ± 35.84	163.1 ± 48.71	160.0 ± 28.57	160.6 ± 31.56	0.992
Creatinine (mg/dl)	75.6 ± 34.37	81.8 ± 52.12	83.1 ± 80.41	81.0 ± 42.15	0.004
BUN (mmol/L)	5.1 ± 1.99	5.5 ± 2.12	5.5 ± 2.55	5.6 ± 2.19	<0.001
TB (μmol/L)	8.2 ± 5.27	8.1 ± 4.99	7.5 ± 4.57	7.8 ± 4.48	0.173
TC (mmol/L)	4.6 ± 1.05	4.9 ± 1.03	4.8 ± 1.09	4.8 ± 1.08	<0.001
TG (mmol/L)	1.2 ± 0.78	1.6 ± 0.96	1.6 ± 1.02	2.1 ± 1.59	<0.001
LDL-C (mmol/L)	2.7 ± 0.93	2.9 ± 0.93	2.8 ± 0.95	2.9 ± 0.98	0.002
HDL-C (mmol/L)	1.4 ± 0.36	1.3 ± 0.34	1.3 ± 0.30	1.2 ± 0.31	<0.001
UA (μmol/L)	305.8 ± 82.05	333.3 ± 83.53	334.8 ± 85.58	354.6 ± 89.10	<0.001
Ferritin (ng/ml)	119.2 ± 160.67	168.4 ± 227.41	151.8 ± 166.63	180 ± 178.92	<0.001
Serum iron (μmol/L)	15.6 ± 6.70	15.2 ± 6.05	14.7 ± 5.68	14.9 ± 5.73	0.019
UIBC (μmol/L)	43.1 ± 12.08	42.9 ± 11.43	44.3 ± 12.37	43.3 ± 10.45	0.968
TIBC (μmol/L)	58.8 ± 9.52	58.2 ± 9.27	58.9 ± 9.78	58.3 ± 8.66	0.433
TFS (%)	27.3 ± 12.18	27.0 ± 11.80	25.8 ± 11.47	26.2 ± 10.29	0.040
TFR (nmol/L)	40.5 ± 24.16	43.1 ± 32.68	41.5 ± 29.55	39.9 ± 16.97	0.884
Vitamin C (μmol/L)	55.4 ± 28.20	52.8 ± 27.49	51.9 ± 27.07	47.5 ± 26.54	<0.001
**LUTE**					
Probe type					<0.001
M	1,983 (90.7)	224 (74.7)	148 (71.5)	445 (48.1)	
XL	201 (9.3)	75 (25.3)	59 (28.5)	479 (51.9)	
Complete measure times	11.5 ± 2.40	11.6 ± 2.64	11.6 ± 2.71	11.5 ± 2.55	0.866
Attempted measure times	14 ± 4.83	15.7 ± 5.89	15.9 ± 6.35	16.1 ± 6.31	<0.001
E value (kPa)	5.0 ± 3.34	5.6 ± 3.20	5.7 ± 3.46	7 ± 5.41	<0.001
E-IQR	0.7 ± 0.52	0.9 ± 0.81	0.8 ± 0.63	1.1 ± 1.04	<0.001
CAP value (dB/m)	215.8 ± 38.52	281 ± 4.61	295.4 ± 3.27	340.6 ± 28.83	<0.001
CAP-IQR	40.4 ± 20.78	37.8 ± 19.55	38.8 ± 20.69	33.7 ± 19.62	<0.001
**Clinical prediction rules**					
AST/ALT ratio	1.30 ± 0.44	1.12 ± 0.40	1.07 ± 0.37	0.98 ± 0.37	<0.001
APRI	0.27 ± 0.20	0.27 ± 0.14	0.30 ± 0.32	0.31 ± 0.25	<0.001
BARD	2.25 ± 0.76	2.52 ± 0.98	2.45 ± 1.06	2.55 ± 1.12	<0.001
FIB-4	0.95 ± 0.77	1.09 ± 0.69	1.29 ± 2.67	1.14 ± 0.79	<0.001
NAFLD fibrosis score	−2.19 ± 1.67	−1.4 ± 1.63	−1.38 ± 1.64	−0.96 ± 1.68	<0.001

CAP, controlled attenuation parameter; PIR, poverty income ratio; SBP, systolic blood pressure; DBP, diastolic blood pressure; BMI, body mass index; WC, waist circumference; HC, hip circumference; WHR, waist-to-hip ratio; MS, metabolic syndrome; ALT, alanine transaminase; AST, aspartate aminotransferase; GGT, gamma-glutamyl transferase; ALP, alkaline phosphatase; FPG, fasting blood glucose; FINS, fasting insulin; HOMA-IR, homeostasis model assessment of insulin resistance; HbA1c, glycohemoglobin; LDH, lactate dehydrogenase; BUN, blood urea nitrogen; TB, total bilirubin; TC, total cholesterol; TG, triglycerides; LDL, low density lipoprotein; HDL, high density lipoprotein; UA, uric acid; UIBC, unsaturated iron binding capacity; TIBC, total iron binding capacity; TFS, transferrin saturation; TFR, transferrin receptor; LUTE, liver ultrasound transient elastography; APRI, AST to platelet ratio; BARD, BMI-AST:ALT-Diabetes; FIB-4, fibrosis index based on 4 regression coefficients.

* Sample weight correction recommended by NHANES was used for data analysis. One-way ANOVA and Rao-Scott χ^2^-test were used for comparison between groups.

### Ferritin and serum Vit C correlated to fibrosis severity

All subjects were also divided into four fibrosis groups according to the degree of fibrosis assessed by the E value. Similar to steatosis, the percentage of men (*P* = 0.049) and age were higher (*P* < 0.001) in more severe fibrosis. Systolic blood pressure (SBP, *P* < 0.001), DBP (*P* = 0.016), body weight, BMI, WC, and WHR, the rates of hypertension, diabetes, hyperlipidemia, hyperuricemia, and MetS, liver injury makers ALT, AST, and GGT levels, glucose metabolic markers, FPG, FINS, HMOA-IR, and HbA1c also increased with the increasing degree of hepatic fibrosis (all *P* < 0.001). Triglyceride increased while HDL-C decreased in more severe hepatic fibrosis group (all *P* < 0.001). Likewise, serum ferritin significantly increased; while Vit C significantly decreased with more severe hepatic fibrosis (all *P* < 0.001) (Table 2).

**Table 2:** Characteristics of the study population according to E values^*^.

	**Categories of liver fibrosis**				***P*-value**
	**F0–F1**	**F2**	**F3**	**F4**	
Patients, *n* (%)	3,296 (92.6)	110 (2.7)	127 (2.4)	81 (2.3)	
**Demographics Data**					
Gender					0.049
Male	1,662 (52.0)	64 (58.6)	79 (61.5)	52 (62.4)	
Female	1,634 (48.0)	46 (41.4)	48 (38.5)	29 (37.6)	
Age (years)	45.1 ± 0.4	55.4 ± 1.7	56.7 ± 1.7	56.3 ± 1.9	<0.001
Ethnicity					0.235
Non-Hispanic White	1,108 (62.0)	28 (52.0)	42 (54.5)	31 (60.9)	
Non-Hispanic Black	726 (11.1)	28 (11.7)	36 (18.5)	10 (7.0)	
Mexican American	441 (8.7)	19 (9.8)	18 (12.8)	13 (9.3)	
Hispanics	286 (6.5)	13 (14.4)	11 (5.1)	12 (8.6)	
Asian	552 (7.1)	15 (6.8)	13 (6.0)	9 (4.5)	
Others	183 (4.6)	7 (5.3)	7 (3.0)	6 (9.7)	
Marital status					0.562
Married	1,415 (50.4)	58 (57.8)	65 (58.8)	39 (53.9)	
Non-married	1,881 (49.6)	52 (42.2)	62 (41.2)	42 (46.1)	
Education					0.001
Less than high school	502 (9.5)	30 (14.7)	26 (13.8)	18 (13.5)	
High school or equivalent	573 (20.8)	20 (29.7)	32 (30.3)	20 (46.6)	
College or AA degree	1,462 (53.3)	51 (49.5)	55 (48.9)	39 (36.9)	
College graduate or above	759 (16.5)	9 (6.1)	14 (6.9)	4 (3.1)	
PIR					0.101
≤ 1.30	841 (18.3)	23 (17.1)	37 (23.5)	26 (23.4)	
1.31–3.50	1,177 (32.4)	38 (38.9)	55 (43.5)	34 (48.3)	
>3.50	859 (39.3)	30 (32.0)	23 (21.9)	15 (18.6)	
Unknown	419 (10.1)	19 (12.0)	12 (11.0)	6 (9.8)	
**Physical examination**					
Smoke status					0.859
Yes	501 (15.0)	17 (13.9)	18 (10.8)	15 (19.5)	
No/Unknown	2,795 (85.0)	93 (86.1)	109 (89.2)	66 (80.5)	
SBP (mmHg)	122.1 ± 0.35	127.5 ± 1.79	128.6 ± 2	130.9 ± 2.27	<0.001
DBP (mmHg)	72.1 ± 0.21	74.7 ± 1.14	73.8 ± 1.35	74.5 ± 1.32	0.016
Pulse (mmHg)	70.1 ± 0.23	70.7 ± 1.26	68.7 ± 1.26	69.2 ± 1.57	0.576
Height (cm)	165.6 ± 0.18	166.9 ± 1.02	168.8 ± 0.95	167.5 ± 1.11	0.001
Weight (kg)	76.3 ± 0.36	93.3 ± 2.45	99.1 ± 2.5	104.5 ± 4.05	<0.001
BMI (kg/m^2^)					<0.001
Mean, SE	27.7 ± 0.11	33.5 ± 0.83	34.8 ± 0.83	36.9 ± 1.25	<0.001
<25	1,237 (37.5)	18 (16.4)	20 (15.7)	9 (11.1)	
≥25	2,059 (62.5)	92 (83.6)	107 (84.3)	72 (88.9)	
WC (cm)	94.9 ± 0.30	110.6 ± 1.79	113.0 ± 1.87	117.3 ± 2.39	<0.001
HC (cm)	102.9 ± 0.24	113.6 ± 1.65	115.7 ± 1.59	116.8 ± 2.23	<0.001
WHR	0.92 ± 0.001	0.97 ± 0.007	0.98 ± 0.008	1.01 ± 0.008	<0.001
Vigorous recreational activities					<0.001
Yes	642 (19.5)	12 (10.9)	13 (10.2)	7 (8.6)	
No/Unknown	2,654 (80.5)	98 (89.1)	114 (89.8)	74 (91.4)	
Moderate recreational activities					0.438
Yes	1,096 (33.3)	39 (35.5)	34 (26.8)	27 (33.3)	
No/Unknown	2,200 (66.7)	71 (64.5)	93 (73.2)	54 (66.7)	
**Comorbidity**					
Hypertension					<0.001
Yes	990 (30.0)	56 (50.9)	77 (60.6)	46 (56.8)	
No/Unknown	2,306 (70.0)	54 (49.1)	50 (39.4)	35 (43.2)	
Diabetes					<0.001
Yes	396 (12.0)	34 (30.9)	47 (37.0)	37 (45.7)	
No/Unknown	2,900 (88.0)	76 (69.1)	80 (63.0)	44 (54.3)	
Hyperlipemia					<0.001
Yes	1,828 (55.5)	84 (76.4)	92 (72.4)	60 (74.1)	
No/Unknown	1,468 (44.5)	26 (23.6)	35 (27.6)	21 (25.9)	
Hyperuricemia					<0.001
Yes	529 (16.0)	25 (22.7)	46 (36.2)	25 (30.9)	
No/Unknown	2,767 (84.0)	85 (77.3)	81 (63.8)	56 (69.1)	
MS					<0.001
Yes	847 (25.7)	58 (52.7)	75 (59.1)	50 (61.7)	
No/Unknown	2,449 (74.3)	52 (47.3)	52 (40.9)	31 (38.3)	
**Laboratory feature**					
Platelet count ( ×10^9^/L)	246.4 ± 1.11	234.1 ± 6.27	234.6 ± 5.38	211.3 ± 8.08	<0.001
Albumin (g/dl)	4.1 ± 0.01	4.0 ± 0.03	4.0 ± 0.03	3.9 ± 0.04	<0.001
ALT (IU/L)	19.9 ± 0.25	27.8 ± 2.01	26.1 ± 1.54	36.3 ± 3.6	<0.001
AST (IU/L)	20.7 ± 0.16	25.3 ± 1.8	25.4 ± 1.45	35.4 ± 3.37	<0.001
GGT (IU/L)	24.3 ± 0.46	34.9 ± 3.09	50.0 ± 6.47	71.1 ± 8.62	<0.001
ALP (IU/L)	95.1 ± 1.04	87.8 ± 3.52	90.1 ± 3.64	97.6 ± 4.41	0.437
FPG (mmol/L)	6.1 ± 0.04	8.0 ± 0.56	6.8 ± 0.25	8.1 ± 0.58	<0.001
FINS (mU/L)	13.3 ± 0.4	18.5 ± 1.56	22.5 ± 4.66	33.7 ± 11.84	<0.001
HOMA-IR	3.8 ± 0.16	6.8 ± 0.82	8.3 ± 2.7	11.1 ± 3.03	<0.001
HbA1c (%)	5.7 ± 0.02	6.5 ± 0.16	6.3 ± 0.11	6.6 ± 0.18	<0.001
LDH (IU/L)	159.6 ± 0.59	162.5 ± 3.38	164.8 ± 3.17	189.4 ± 9.27	<0.001
Creatinine (mg/dl)	76.7 ± 0.64	87.9 ± 8.93	96.9 ± 6.55	84.1 ± 6.52	<0.001
BUN (mmol/L)	5.2 ± 0.04	5.8 ± 0.29	6.2 ± 0.21	5.8 ± 0.27	<0.001
TB (μmol/L)	8.0 ± 0.09	7.7 ± 0.37	8.6 ± 0.41	10.2 ± 0.7	0.001
TC (mmol/L)	4.7 ± 0.02	4.6 ± 0.11	4.7 ± 0.1	4.4 ± 0.12	0.153
TG (mmol/L)	1.5 ± 0.02	2.3 ± 0.23	1.8 ± 0.16	1.8 ± 0.13	<0.001
LDL-C (mmol/L)	2.8 ± 0.02	2.7 ± 0.19	2.7 ± 0.17	2.6 ± 0.14	0.412
HDL-C (mmol/L)	1.4 ± 0.01	1.2 ± 0.03	1.3 ± 0.04	1.2 ± 0.04	<0.001
UA (μmol/L)	319.3 ± 1.48	335.3 ± 8.05	368.6 ± 10.23	350.3 ± 10.08	<0.001
Ferritin (ng/ml)	132.5 ± 2.76	206.4 ± 24.57	200.5 ± 20.9	290.1 ± 39.12	<0.001
Serum iron (μmol/L)	15.4 ± 0.11	14.4 ± 0.52	14.5 ± 0.58	16.4 ± 0.9	0.076
UIBC (μmol/L)	43.3 ± 0.20	43.2 ± 1.05	42.4 ± 0.89	42.4 ± 1.68	0.779
TIBC (μmol/L)	58.7 ± 0.16	57.7 ± 0.90	56.9 ± 0.76	59.1 ± 1.16	0.126
TFS (%)	27 ± 0.20	25.6 ± 0.98	25.9 ± 0.93	28.9 ± 1.93	0.199
TFR (nmol/L)	40.5 ± 0.42	38.8 ± 1.37	41.2 ± 1.10	44.0 ± 3.32	0.503
Vitamin C (μmol/L)	53.8 ± 0.48	48.7 ± 2.50	41.3 ± 2.3	44.3 ± 3.41	<0.001
**LUTE**					
Probe type					<0.001
M	2,647 (80.3)	64 (58.2)	65 (51.2)	24 (29.6)	
XL	649 (19.7)	46 (41.8)	62 (48.8)	57 (70.4)	
Complete measure times	11.5 ± 0.04	11.9 ± 0.31	12.2 ± 0.32	12.5 ± 0.40	0.044
Attempted measure times	14.5 ± 0.09	16.8 ± 0.64	18.0 ± 0.63	17.6 ± 0.69	<0.001
E value (kPa)	4.9 ± 0.02	8.7 ± 0.04	11.1 ± 0.09	24.5 ± 1.7	<0.001
E-IQR	0.7 ± 0.01	1.3 ± 0.05	1.9 ± 0.07	3.9 ± 0.25	<0.001
CAP value (dB/m)	252.8 ± 1.07	305.8 ± 5.87	305.6 ± 6.02	310.5 ± 6.87	<0.001
CAP-IQR	38.5 ± 0.35	33.4 ± 1.84	35.7 ± 2.1	41.1 ± 3.17	0.016
**Clinical prediction rules**					
AST/ALT ratio	1.2 ± 0.01	1.04 ± 0.03	1.09 ± 0.04	1.07 ± 0.04	<0.001
APRI	0.27 ± 0.002	0.35 ± 0.04	0.36 ± 0.03	0.64 ± 0.08	<0.001
BARD	2.32 ± 0.02	2.66 ± 0.10	2.76 ± 0.09	2.98 ± 0.11	<0.001
FIB-4	0.99 ± 0.02	1.3 ± 0.08	1.42 ± 0.10	2.03 ± 0.2	<0.001
NAFLD fibrosis score	−1.9 ± 0.03	−0.68 ± 0.16	−0.46 ± 0.15	0.21 ± 0.2	<0.001

CAP, controlled attenuation parameter; PIR, poverty income ratio; SBP, systolic blood pressure; DBP, diastolic blood pressure; BMI, body mass index; WC, waist circumference; HC, hip circumference; WHR, waist-to-hip ratio; MS, metabolic syndrome; ALT, alanine transaminase; AST, aspartate aminotransferase; GGT, gamma-glutamyl transferase; ALP, alkaline phosphatase; FPG, fasting blood glucose; FINS, fasting insulin; HOMA-IR, homeostasis model assessment of insulin resistance; HbA1c, glycohemoglobin; LDH, lactate dehydrogenase; BUN, blood urea nitrogen; TB, total bilirubin; TC, total cholesterol; TG, triglycerides; LDL, low density lipoprotein; HDL, high density lipoprotein; UA, uric acid; UIBC, unsaturated iron binding capacity; TIBC, total iron binding capacity; TFS, transferrin saturation; TFR, transferrin receptor; LUTE, liver ultrasound transient elastography; APRI, AST to platelet ratio; BARD, BMI-AST:ALT-Diabetes; FIB-4, fibrosis index based on 4 regression coefficients.

* Sample weight correction recommended by NHANES was used for data analysis. One-way ANOVA and Rao-Scott χ^2^-test were used for comparison between groups.

### Ferritin and serum Vit C correlated to other metabolic disorders

Serum ferritin levels were significantly higher in subjects with hypertension, diabetes, dyslipidemia, hyperuricemia, or MetS than in subjects without corresponding metabolic disorders (all *P* < 0.001). Vitamin C was significantly lower in in subjects with diabetes, dyslipidemia, hyperuricemia, or MetS than subjects without corresponding metabolic disorders (all *P* < 0.001) (Supplementary Figure 2).

### Association between dietary intake of Vit C and risk of NAFLD

**Figure 1:**
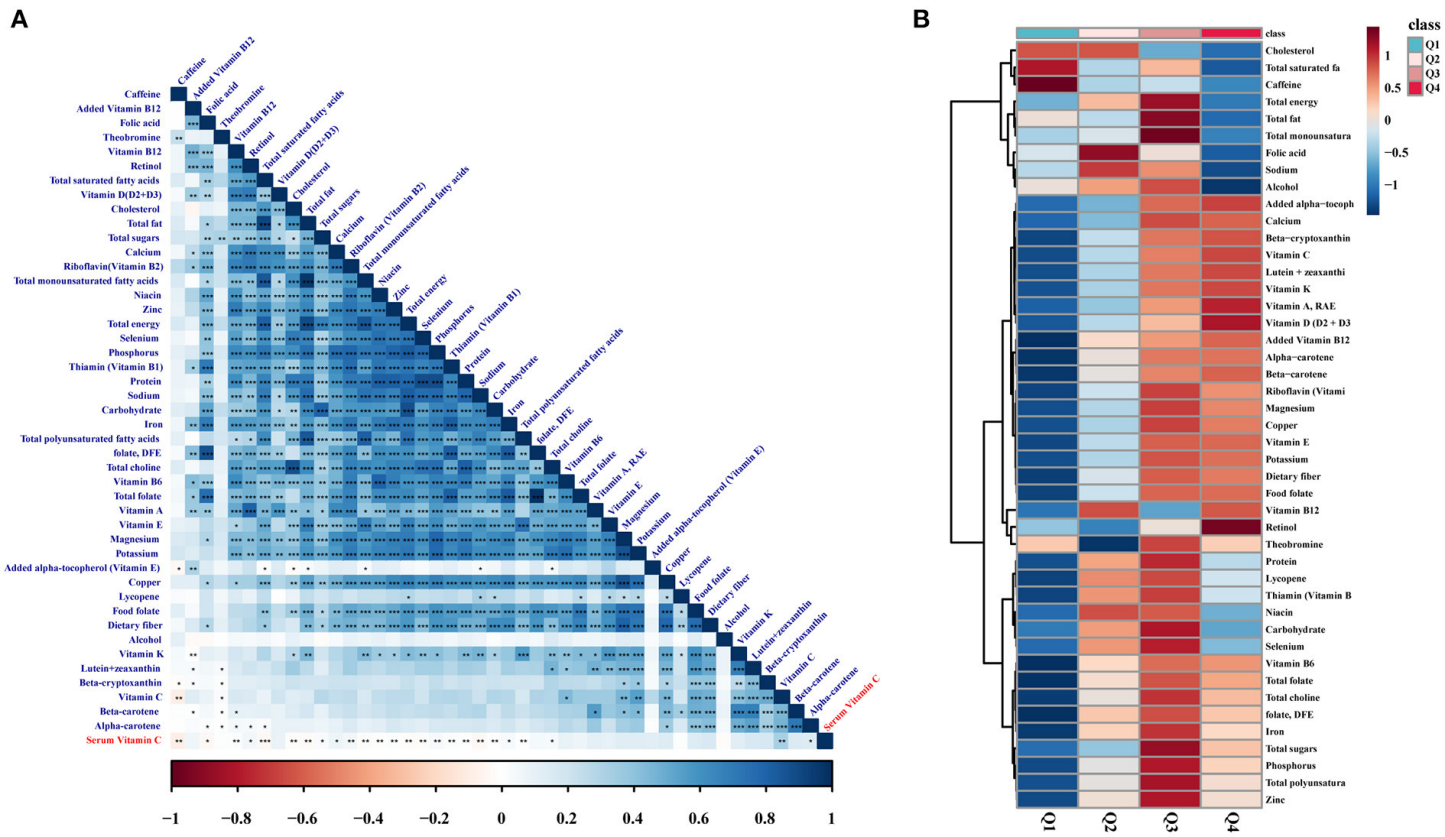
Association between serum Vit C and dietary intake of nutrients. **(A)** Pearson's correlation heatmap between serum Vit C and dietary intake of nutrients. **(B)** Average heatmap of nutrient intake between different Vit C intake groups. *p < 0.05, **p < 0.01, ***p < 0.001.

Based on the results above about serum Vit C, we were wondering about the effect of Vit C dietary intake on NAFLD. Therefore, we analyzed the dietary data of the enrolled samples. However, the risk of NAFLD was not reduced in the high-dose Vit C intake group (Q4, Supplementary Table 1). The correlation analysis showed serum Vit C levels were not only correlated with Vit C intake, but also with intake of nutrients including fat, saturated fatty acids, sugar, caffeine, iron, and Vitamin B12 (Figure 1A). Further analysis revealed that although Vit C intake was high in participants, the intake of other nutrients including sugar, iron, and saturated fatty acids, which would lower serum Vit C were also increased (Figure 1B). Additionally, the metabolic disorder in the liver diseases complicates the regulation of Vit C. This explains why the high dose of Vit C intake in the study cohort fails to produce a protective effect against NAFLD and cirrhosis. Therefore, serum Vit C level is more reasonable and reliable for clinical analysis and guidance for NAFLD patients.

### Association of serum Vit C and ferritin with hepatic steatosis assessed by logistic regression and RCS models

**Figure 2:**
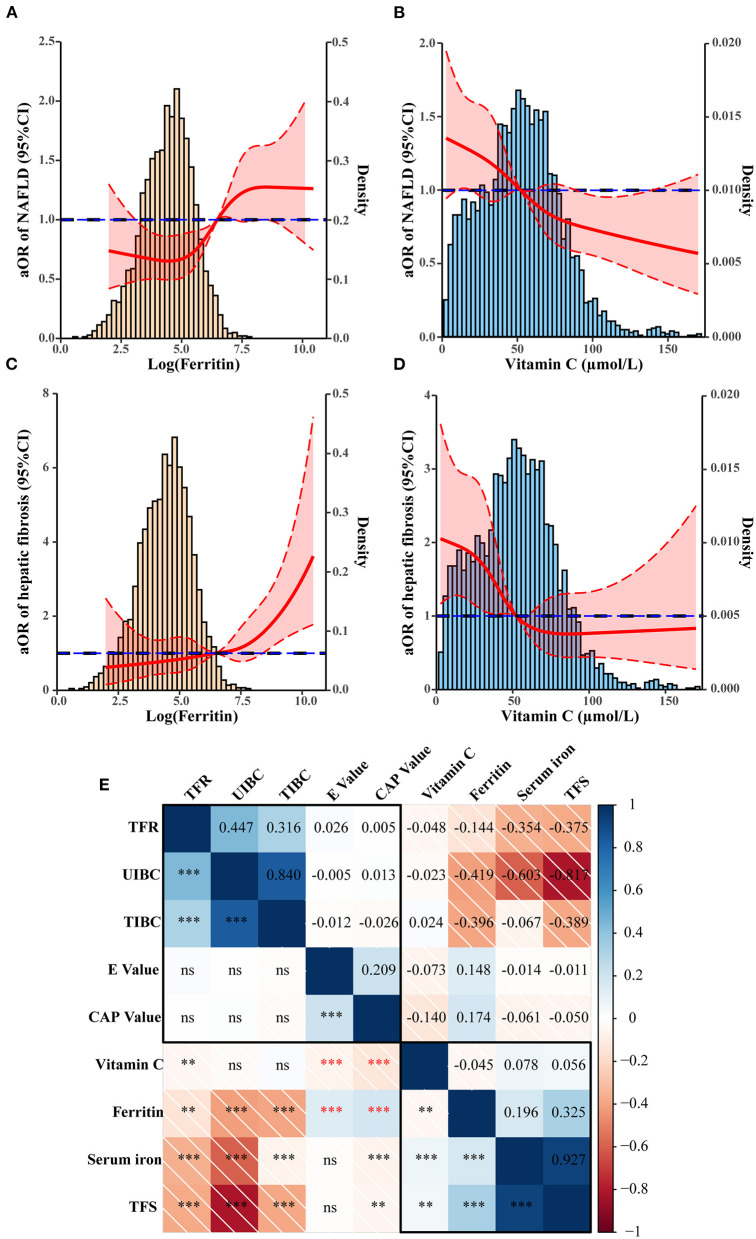
Association between Vit C, ferritin, and NAFLD. **(A,B)** Restricted cubic spline (RCS) showing the relationship between Ferritin and Vitamin C with adjusted odds ratio of NAFLD (solid line). **(A,C)** Ferritin, **(B,D)** Vitamin C. The 95% confidence interval is indicated by the dashed line area. **(E)** Correlation between iron metabolism, Vitamin C, and liver ultrasound transient elastography value. **p < 0.01, ***p < 0.001.

To explore the roles of ferritin and Vit C on hepatic steatosis, we adjusted for age, sex, race, education level, marital status, and PIR, increased serum ferritin was a risk factor for hepatic steatosis (1.49 [1.18–1.88], 1.81 [1.43–2.30], 2.15 [1.68–2.74], all *P* < 0.001), while increased Vit C was a protective factor for NAFLD (0.83 [0.68–1.01], 0.63 [1.43–2.30], 0.47 [0.38–0.58], all *P* < 0.001). Adjusted for the above parameters plus BMI, smoking status, and vigorous recreational activities, increased serum ferritin was still a risk factor for NAFLD (1.59 [1.24–2.05], 1.71 [1.33–2.20], 2.02 [1.56–2.62], all *P* < 0.001), while increased Vit C was a protective factor for NAFLD (0.82 [0.66–1.02], 0.65 [0.52–0.81], 0.55 [0.44–0.70], all *P* < 0.001). Adjusted for hypertension, diabetes, kidney failure, hyperlipemia, hyperuricemia, and MetS, increased serum ferritin was still a risk factor for NAFLD (1.52 [1.17–1.96], 1.72 [1.33–2.23], 1.95 [1.49–2.55], all *P* < 0.001), while increased Vit C was a protective factor for NAFLD (0.82 [0.66–1.03], 0.70 [0.56–0.88], 0.61 [0.48–0.77], all *P* < 0.001), as presented in Table 3. To avoid the bias caused by any possible leverage values, we have also used the RCS model to fit the non-linear relationship between Vit C, ferritin, and NAFLD, we found that the odds ratio of NAFLD increased significantly with increasing serum ferritin after adjusting for covariates (*P* < 0.001, Figure 2A), while serum Vit C showed the opposite trend (*P* < 0.001, Figure 2B).

**Table 3:** Adjusted odds ratios of the association between the serum ferritin, Vitamin C, and non-alcoholic fatty liver disease in NHANES^*a*^.

**Characteristic**	***n* (%)**	**A model**		**B model**		**C model**	
		**AOR (95%CI)**	***P***	**AOR (95%CI)**	***P***	**AOR (95%CI)**	***P***
Serum ferritin (ng/ml)			<0.001		<0.001		<0.001
Q1 (1.04–41.60)	905 (25.0)	1.00 (Reference)		1.00 (Reference)		1.00 (Reference)	
Q2 (41.61–89.20)	902 (25.0)	1.49 (1.18–1.88)	<0.001	1.59 (1.24–2.05)	<0.001	1.52 (1.17–1.96)	0.001
Q3 (89.21–176.0)	904 (25.0)	1.81 (1.43–2.30)	<0.001	1.71 (1.33–2.20)	<0.001	1.72 (1.33–2.23)	<0.001
Q4 (176.1–2,430)	903 (25.0)	2.15 (1.68–2.74)	<0.001	2.02 (1.56–2.62)	<0.001	1.95 (1.49–2.55)	<0.001
Vitamin C (μmol/L)			<0.001		<0.001		<0.001
Q1 (1.20–33.70)	904 (25.0)	1.00 (Reference)		1.00 (Reference)		1.00 (Reference)	
Q2 (33.71–52.20)	905 (25.0)	0.83 (0.68–1.01)	0.068	0.82 (0.66–1.02)	0.079	0.82 (0.66–1.03)	0.086
Q3 (52.21–69.30)	913 (25.3)	0.63 (0.52–0.78)	<0.001	0.65 (0.52–0.81)	<0.001	0.70 (0.56–0.88)	0.002
Q4 (69.31–359)	892 (24.7)	0.47 (0.38–0.58)	<0.001	0.55 (0.44–0.70)	<0.001	0.61 (0.48–0.77)	<0.001

NHANES, National Health and Nutrition Examination Survey; AOR, adjusted odds ratio; Q, quartile.

^*a*^Adjusted covariates: A model: age, gender, race, education levels, marital status, and household poverty income ratio (PIR); B model: A model plus body mass index (BMI), smoking status, vigorous recreational activities; C model: B model plus hypertension, diabetes, kidney failure, hyperlipemia, hyperuricemia, and metabolic syndrome.

### Association of serum Vit C and ferritin with hepatic fibrosis assessed by logistic regression and RCS models

Similarly, adjusted for confounding covariates, the highest ferritin group (176.1–243.0 ng/ml) was a risk factor for hepatic fibrosis (1.83 [1.12–2.99], *P* = 0.016), while the highest serum Vit C group (69.31–359 μmol/L) was a protective factor for hepatic fibrosis (0.39 [0.24–0.61], *P* < 0.001) (Table 4). By using the RCS model we found that the odds ratio of hepatic fibrosis increased significantly with increasing serum ferritin after adjusting for covariates (*P* < 0.001, Figure 2C), while elevated serum Vit C significantly decreased the odds ratio of hepatic fibrosis (*P* < 0.001, Figure 2D).

**Table 4:** Adjusted odds ratios of the association between the serum ferritin, Vitamin C, and hepatic fibrosis (F3–F4) in NHANES^*a*^.

**Characteristic**	***n* (%)**	**A model**		**B model**		**C model**	
		**AOR (95%CI)**	***P***	**AOR (95%CI)**	***P***	**AOR (95%CI)**	***P***
Serum ferritin (ng/ml)			0.006		0.007		0.012
Q1 (1.04–41.60)	905 (25.0)	1.00 (Reference)		1.00 (Reference)		1.00 (Reference)	
Q2 (41.61–89.20)	902 (25.0)	1.21 (0.74–2.00)	0.449	1.23 (0.74–2.03)	0.427	1.20 (0.72–1.99)	0.492
Q3 (89.21–176.0)	904 (25.0)	1.06 (0.64–1.76)	0.825	1.01 (0.61–1.68)	0.963	1.04 (0.62–1.74)	0.874
Q4 (176.1–243.0)	903 (25.0)	1.89 (1.17–3.05)	0.010	1.83 (1.13–2.97)	0.014	1.83 (1.12–2.99)	0.016
Vitamin C (μmol/L)			<0.001		<0.001		<0.001
Q1 (1.20–33.70)	904 (25.0)	1.00 (Reference)		1.00 (Reference)		1.00 (Reference)	
Q2 (33.71–52.20)	905 (25.0)	0.67 (0.47–0.95)	0.024	0.65 (0.45–0.93)	0.018	0.67 (0.46–0.96)	0.030
Q3 (52.21–69.30)	913 (25.3)	0.40 (0.27–0.61)	<0.001	0.40 (0.26–0.61)	<0.001	0.44 (0.29–0.68)	<0.001
Q4 (69.31–359)	892 (24.7)	0.32 (0.20–0.50)	<0.001	0.34 (0.22–0.54)	<0.001	0.39 (0.24–0.61)	<0.001

NHANES, National Health and Nutrition Examination Survey; AOR, adjusted odds ratio; Q, quartile.

^*a*^Adjusted covariates: A model: age, gender, race, education levels, marital status, and household poverty income ratio (PIR); B model: A model plus body mass index (BMI), smoking status, vigorous recreational activities; C model: B model plus hypertension, diabetes, kidney failure, hyperlipemia, hyperuricemia, and metabolic syndrome.

### Ferritin mediates the impact of Vit C and non-alcoholic liver disease

**Figure 3:**
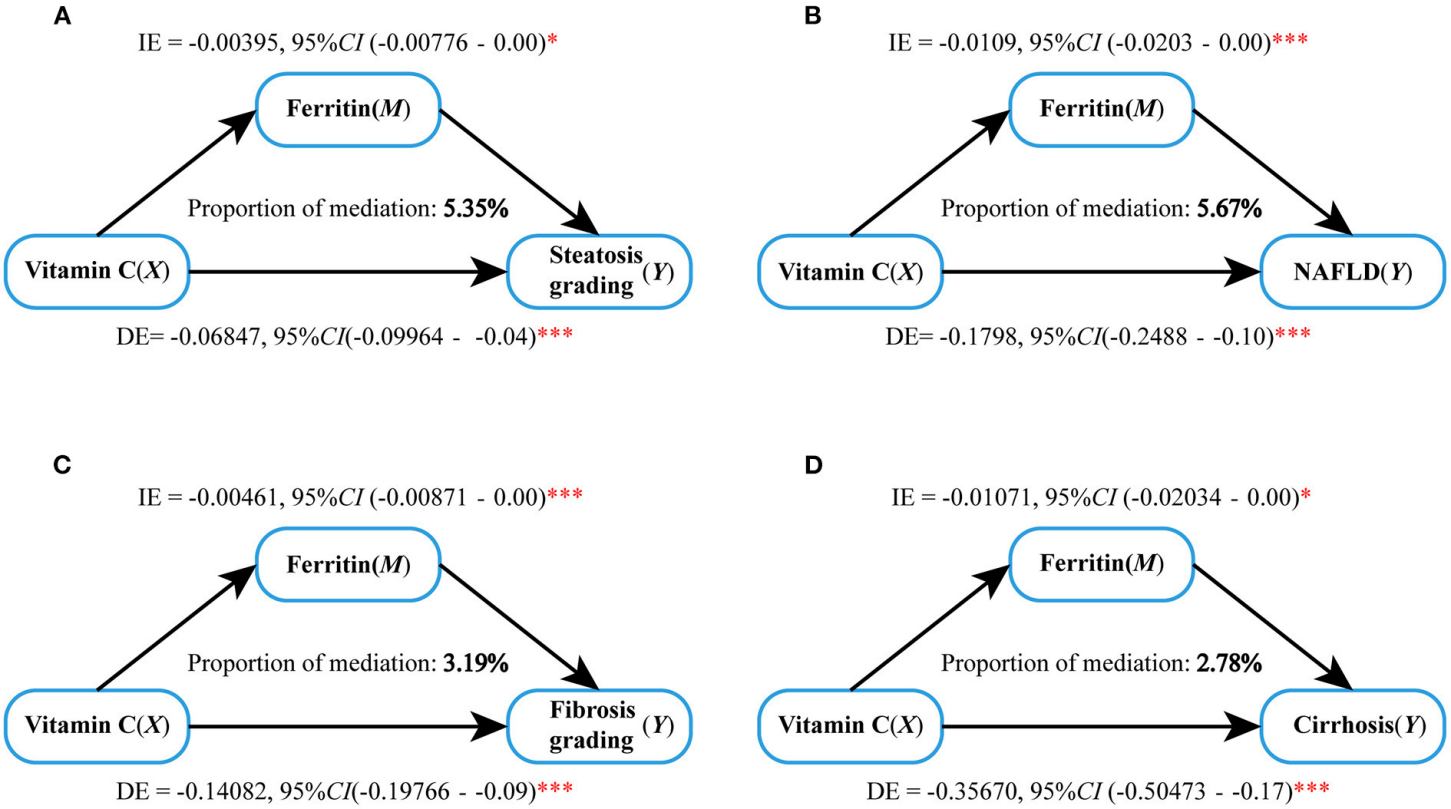
Mediation analysis of serum ferritin on the interaction between Vitamin C and prevalence of NAFLD and cirrhosis. **(A)** Steatosis grading, **(B)** NAFLD, **(C)** fibrosis grading, and **(D)** cirrhosis. *p < 0.05, ***p < 0.001.

According to the correlation analysis, serum Vit C was negatively associated with E and CAP values (*r* = −0.073, *P* < 0.001; *r* = −0.140, *P* < 0.001), while ferritin was positively associated with the E and CAP values (*r* = 0.148, *P* < 0.001; *r* = −0.174, *P* < 0.001). We further raised the question that whether there was a connection between Vit C and ferritin. Surprisingly, Vit C was found negatively associated with ferritin levels (*r* = −0.045, *P* < 0.01) (Figure 2E). Mediation analysis was performed to verify whether ferritin partly mediates the impact of Vit C on NAFLD. The results showed that Vit C may act on NAFLD by acting on ferritin, as shown in Figure 3. Ferritin partly mediated efficacy account for 5.35% of the association of Vit C and steatosis grading (IE = −0.00395, 95% CI: −0.00776 to 0.00; DE = −0.06847, 95% CI: =-0.09964 to −0.04, Figure 3A), while account for 5.67% of the association of Vit C and NAFLD (IE = −0.0109, 95% CI: −0.0203 to 0.00; DE = −0.1798, 95% CI: −0.2488 to −0.10, Figure 3B), account for 3.19 of the association of Vit C and fibrosis grading (IE = −0.00461, 95% CI: −0.00871 to 0.00; DE =-0.14082, 95% CI: −0.19766 to −0.09, Figure 3C), and account for 2.78 of the association of Vit C and cirrhosis (IE = −0.01071, 95% CI: −0.002034 to 0.00; DE = −0.35670, 95% CI: −0.50473 to −0.17, Figure 3D).

### Ascorbic acid (vitamin C) alleviated steatosis *in vitro*

**Figure 4:**
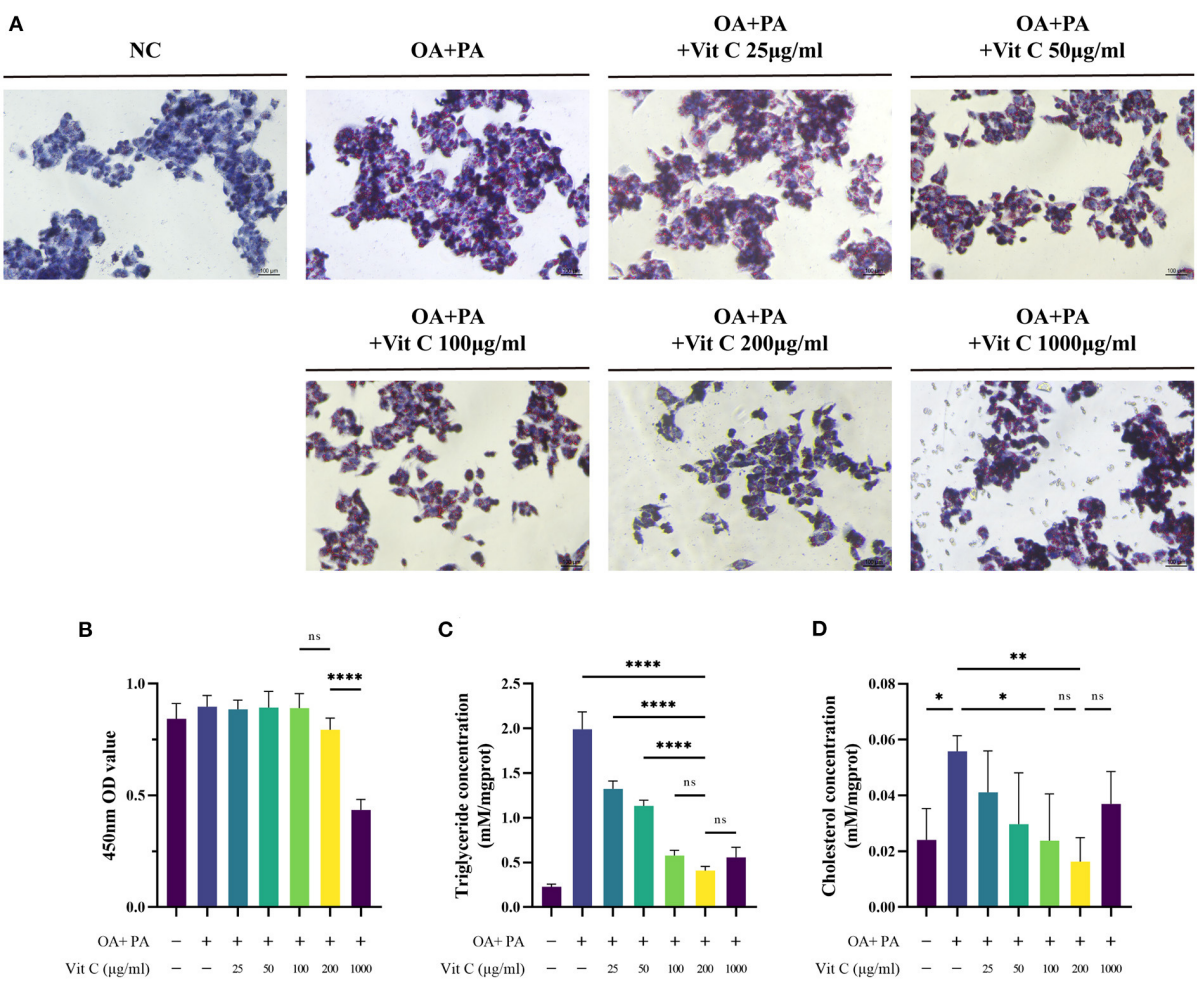
Adding ascorbic acid (Vitamin C) to HepG2 cells in PA/OA culture reduces intracellular lipid deposition. **(A)** HEPG2 cells stained with Oil Red O, **(B)** CCK-8 assay for cell viability (*n* = 5). **(C,D)** Triglyceride and cholesterol concentrations in the cells were detected after 24 h (*n* = 4). Values were presented as means ± SEM. *p < 0.05, **p < 0.01, ****p < 0.0001.

To verify the protective effect of Vit C on NAFLD, we induced lipid accumulation with PA/OA in liver cell line hepG2 and treated it with Vitamin C. We found that intracellular lipid deposition gradually decreased with increasing Vit C concentration in the HepG2 cells (Figure 4A) and 200 μg/ml concentration of Vit C significantly suppressed the elevated levels of intracellular cholesterol and triglycerides (Figures 4C,D). However, extremely high concentrations of Vit C levels did not provide further protection against cellular lipid deposition, which may be due to the inhibitory effect on cellular activity, that is adverse effects or toxicity (Figure 4B). This is also consistent with the published animal study (4).

### Vitamin C maintains iron homeostasis by inhibiting PA/OA induced ferritin upregulation and LIP induction

**Figure 5:**
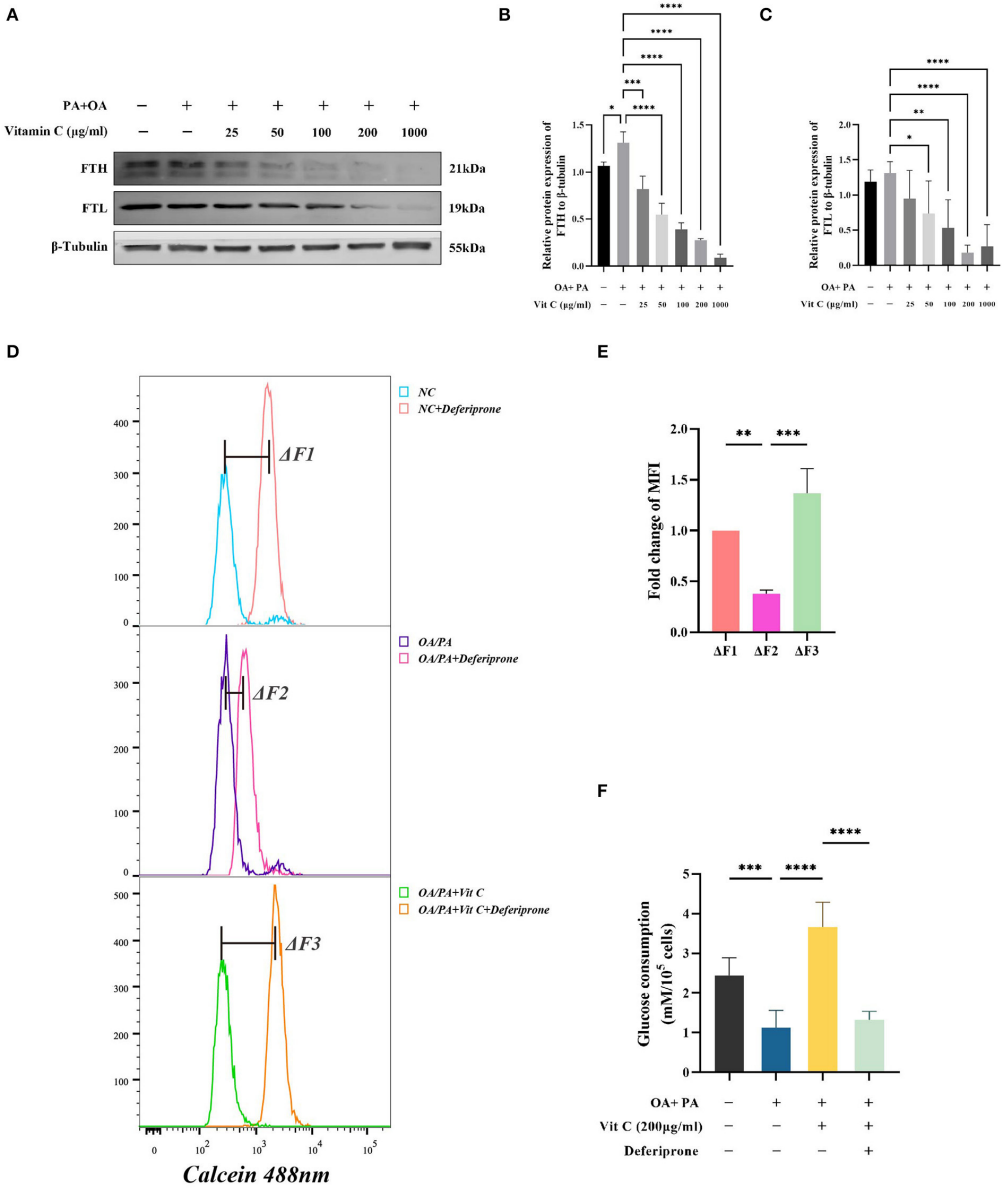
Vitamin C effects on intracellular ferritin and LIP. **(A–C)** The protein levels of FTL and FTH in the HepG2 were determined by Western blot. **(D)** Vitamin C increased the labile iron pool in HepG2 cells, **(E)** Quantification of the levels of labile iron pool (*n* = 3), and **(F)** Glucose concentration in the medium was measured after 24 h treatment of HepG2 with 200 μg/ml Vitamin C and 100 μM deferiprone, respectively (*n* = 6). Values were presented as means ± SEM. *p < 0.05, **p < 0.01, ***p < 0.001, ****p < 0.0001.

To answer whether Vitamin C treatment has an influence on ferritin, ferritin levels were detected with or without Vitamin C stimulation in HepG2 cells. We observed that FTH levels were elevated by about 23% after the addition of PA/OA and Vit C inhibited ferritin light and heavy chain levels in a dose dependent manner (Figures 5A–C). We also noticed a significant reduction of the LIP within HepG2 cells after PA/OA addition. The reduction of LIP due to palmitic and OA could be restored by supplementation of Vitamin C (Figures 5D,E). Moreover, Vit C reversed the metabolic dysfunction caused by lipotoxic substances and improved the cellular uptake of glucose, while its effect may be mediated by influencing intracellular LIP levels (Figure 5F).

## Discussion

Non-alcoholic fatty liver disease, which is tightly linked to MetS, has emerged as a leading cause of chronic liver disease worldwide and is characterized by a wide spectrum of hepatic changes. It can also progress to liver fibrosis and cirrhosis (27). One of the most widely recognized theories of NAFLD is the “two-hit hypothesis,” which developed into a “multiple parallel hit hypothesis” (28). Iron overload syndrome, characterized by increased ferritin levels, is common in patients with NAFLD (12). Serum ferritin is associated with liver fat, as measured using MRI (12). A cross-sectional study included 349 subjects who showed significantly higher serum ferritin levels in the NAFLD group (*P* < 0.001) (13). As a potent pro-oxidant, iron overload can affect major tissues and organs involved in glucose-lipid metabolism, such as the liver (10). Therefore, we explored the status of iron metabolism in NAFLD and its association with hepatic steatosis and fibrosis. Corresponding to previous studies, serum ferritin levels are significantly increased with a higher server level of hepatic steatosis. In obese female patients, ferritin levels were positively associated with serum levels of stem cell growth factor-beta, a growth factor that induces M1 polarization in adipose tissue-infiltrated macrophages (29). Moreover, ferritin, as a non-invasive biomarker, is associated with hepatic fibrosis (30). Elevated serum ferritin levels are associated with hepatic fibrosis in virus-infected and hemochromatosis patients (31–33).

Increased ferritin levels are observed in NAFLD patients, which is also significantly associated with liver fibrosis in men and women in a total of 25,597 participants from the Korean National Health and Nutritional Examination Surveys 2007–2012 (34). A total of 468 patients with biopsy-proven NAFLD from two European centers also indicated serum ferritin levels with worsening fibrosis up to the pre-cirrhotic stage (35). Our results also verified the relationship between ferritin and hepatic fibrosis. Overall, iron metabolism not only affects hepatic steatosis but also impacts hepatic fibrosis.

Many therapeutic approaches have been proposed for the treatment of NAFLD. The most promising of these are those with antioxidant effects, including VE and polyphenols, which reduce lipogenesis (27). Insufficient evidence has proven that Vit C affects patients with NAFLD without well-designed randomized controlled studies (RCTs) (27). A recently published double-blind RCT showed that 12 weeks of Vit C supplementation, especially 1,000 mg/day, improved liver health in patients with NAFLD (7). Studies on humans have supported the association between inadequate Vit C intake and NAFLD (5). A cross-sectional study including 789 patients showed that the upper tertile of Vit C intake/1,000 Kcal was associated with lower odds of NAFLD and NASH (*OR* = 0.68, 0.47–0.99, *P* = 0.045; *OR* = 0.57, 0.38–0.84, *P* = 0.004, respectively), indicating that Vit C may play a protective role in NAFLD, even in NASH (5). A cross-sectional study that included 3,471 subjects showed a significant inverse association between dietary Vit C intake and NAFLD (6). Consistent with a previous study, our study showed that Vit C status is closely related to both hepatic steatosis and hepatic fibrosis. Vitamin C concentration may be a protective factor against NAFLD.

Vitamin C is a highly effective antioxidant that donates electrons, thus protecting important biomolecules such as lipids and nucleic acids from oxidative damage (36). As a cofactor of a family of biosynthetic and gene regulatory monooxygenase and dioxygenase enzymes (37, 38), Vit C participates in many functions in the prevention of disease. An animal study of mice showed the therapeutic effects of Vit C administration on NAFLD (39). A mechanistic study in rats revealed a marked decrease in the histological alteration of fatty liver induced by a choline-deficient diet due to reduced oxidative stress (8). C57BL/6J mice administered Vit C proved that Vit C seems to suppress HFD-induced NAFLD in part through activation of peroxisome proliferator-activated receptor α (PPARα) (9). Vitamin C, a cofactor of the enzyme 7-α-hydroxylase, which is the rate-limiting enzyme in bile acid synthesis, may promote the conversion of cholesterol into bile acid (40). Vitamin C improves lipid metabolism by increasing LDL-C uptake from the circulation, eventually lowering LDL-C plasma levels, and inhibiting the oxidation of LDL-C, facilitating its binding to LDL receptors on hepatocytes (40, 41). Thus, improved lipid metabolism may facilitate NAFLD. However, in our cohort we did not find a reduced risk of NAFLD with high dose Vit C intake. This may not only be related to individual differences in Vit C absorption and excretion, but more importantly, the high inflammation and hepatic iron accumulation in NAFLD patients counteract the reducing function of Vit C.

However, another study established a Vit C-deficient mouse model to investigate Vit C on lipid metabolism, and the results showed that Vit C deficiency may inhibit *de novo* lipogenesis through impaired sterol regulatory element-binding protein-1c activation (42). Therefore, it is necessary to explore the underlying mechanisms of Vit C in NAFLD. No study has explored the relationship between Vit C and ferritin levels in NAFLD. Only a few studies have investigated the interaction between Vit C and ferritin, indicating that they crosstalk with each other (43, 44). We explored whether the relationship between Vit C and NAFLD is partly mediated by iron metabolism. Correlation analysis revealed a negative relationship between Vit C and ferritin levels in our study. Further mediation analysis revealed that the impact of Vit C on NAFLD is partly mediated by ferritin. The *in vitro* results also showed that Vit C inhibited intracellular ferritin and restored the cellular LIP and glucose metabolism disorders caused by fatty acid accumulation. Therefore, it is inferred that the treatment of NAFLD with Vit C, not only hepatic steatosis but also the progression of fibrosis, may be partly mediated by ferritin.

Undeniably our study still has some limitations. Firstly, our study is based on cross-sectional data from NHANES and cannot analyze the causal relationship between ferritin, Vit C and NAFLD. Secondly, NHANES only has open access to LUTE data for the 2017–2018 cycle, so the sample included in the study is limited. Third, our *in vitro* experiments were unable to elucidate the specific mechanisms by which Vitamin C regulates ferritin and alters intracellular kinetics of iron. Last but not least, longitudinal studies or animal model experiments are needed to validate the ameliorative effect of Vitamin C on NAFLD.

## Conclusion

Our study indicated that increased ferritin is a risk factor while increased Vit C is a protective factor for NAFLD. This may provide evidence for the benefit of Vit C supplementation for hepatic steatosis and fibrosis in individuals. Additionally, the impact of Vit C on NAFLD may be partly mediated by iron metabolism.

## Data availability statement

The raw data supporting the conclusions of this article will be made available by the authors, without undue reservation.

## Ethics statement

Ethical review and approval was not required for the study on human participants in accordance with the local legislation and institutional requirements. The patients/participants provided their written informed consent to participate in this study.

## Author contributions

XW: conceptualization, supporting, funding acquisition, supervision, and writing—review and editing. ZH: writing—original draft and writing—review and editing. YL: formal analysis and writing—review and editing. BM: formal analysis and writing—original draft. SL: writing—review and editing.

## Funding

This research was supported by the Climbing Talent Program of Shanghai Tenth People's Hospital (2021SYPDRC047).

## Conflict of interest

The authors declare that the research was conducted in the absence of any commercial or financial relationships that could be construed as a potential conflict of interest.

## Publisher's note

All claims expressed in this article are solely those of the authors and do not necessarily represent those of their affiliated organizations, or those of the publisher, the editors and the reviewers. Any product that may be evaluated in this article, or claim that may be made by its manufacturer, is not guaranteed or endorsed by the publisher.
