# Supplementary figures and images for: Iron metabolism mediates the relationship between Vitamin C and hepatic steatosis and fibrosis in NAFLD
Source: Front Nutr. 2022 Sep 8;9:952056. doi: 10.3389/fnut.2022.952056 (PMC9494736; doi:10.3389/fnut.2022.952056)

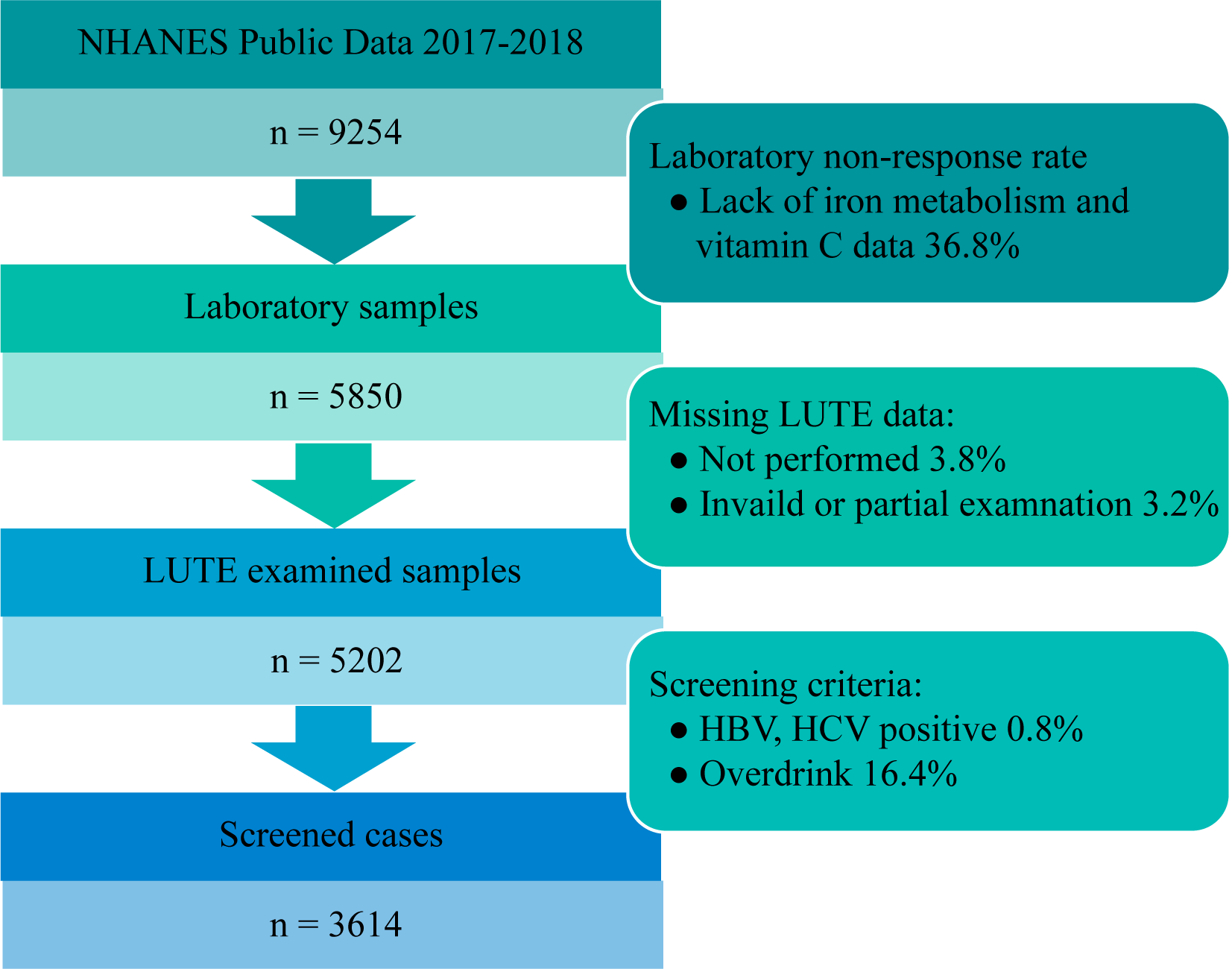

Supplement: Supplementary file 1 [file Image_1.TIF]

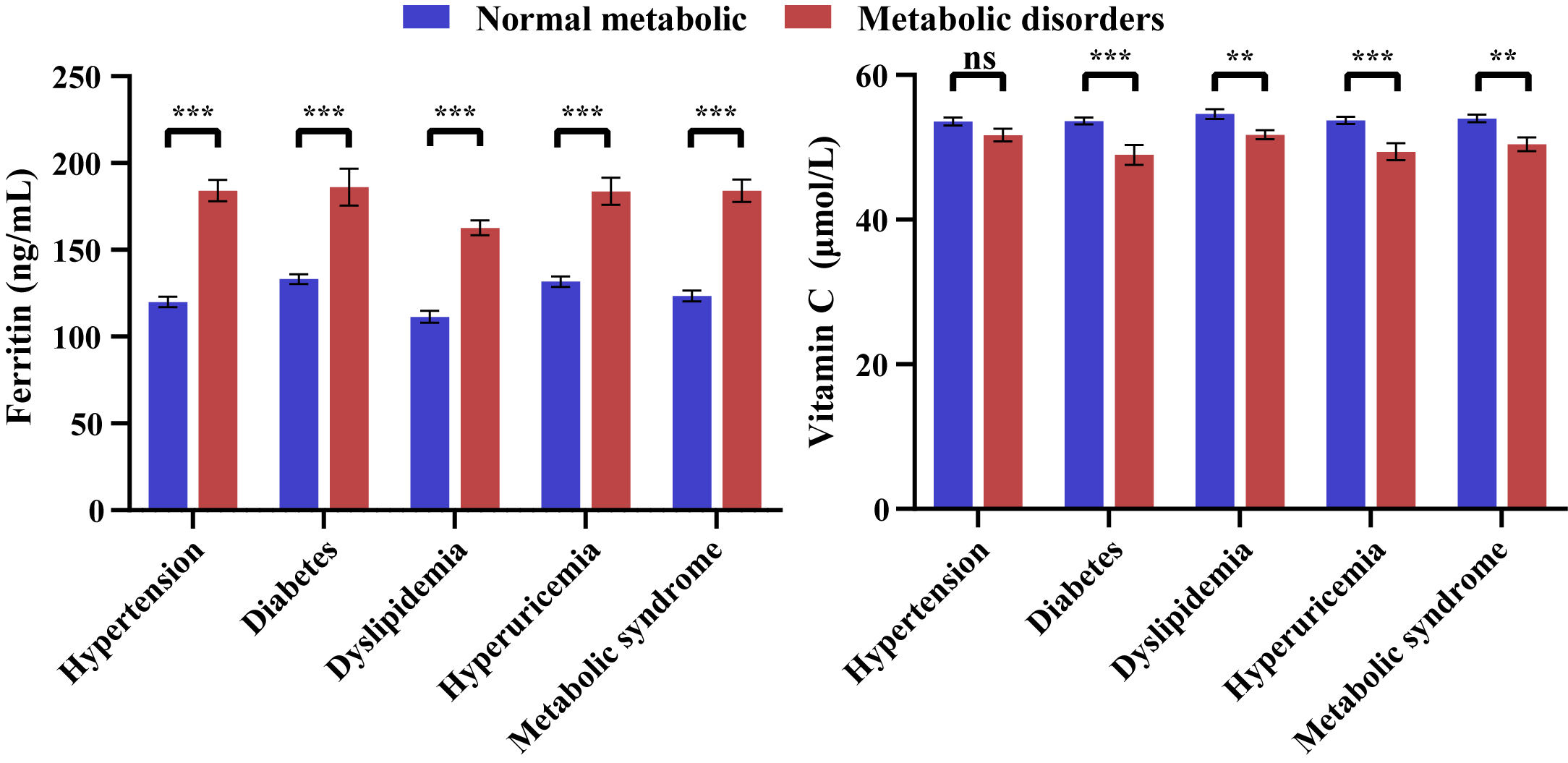

Supplement: Supplementary file 2 [file Image_2.TIF]
