# Supplementary material for: Iron metabolism mediates the relationship between Vitamin C and hepatic steatosis and fibrosis in NAFLD
Source: Front Nutr. 2022 Sep 8;9:952056. doi: 10.3389/fnut.2022.952056 (PMC9494736; doi:10.3389/fnut.2022.952056)
Supplement: Supplementary file 3 [file Table_1.docx]

Table S1. Adjusted odds ratios of the association between the vitamin C intake and NAFLD, cirrhosis in NHANES 2017-2018.

| Vitamin C intake group (mg/day) | A model | | B model | | C model | |
| --- | --- | --- | --- | --- | --- | --- |
|  | AOR (95%CI) | P | AOR (95%CI) | P | AOR (95%CI) | P |
| **NAFLD** |  | 0.100 |  | 0.117 |  | 0.144 |
| Q1(0.00-27.15) | 1.00(reference) |  | 1.00(reference) |  | 1.00(reference) |  |
| Q2(27.16-56.70) | 1.22(0.97-1.54) | 0.087 | 1.29(1.01-1.65) | 0.040 | 1.31(1.01-1.68) | 0.039 |
| Q3(56.71-104.55) | 0.99(0.79-1.26) | 0.983 | 0.99(0.78-1.28) | 0.977 | 1.02(0.79-1.31) | 0.902 |
| Q4(104.56-741.65) | 0.93(0.74-1.17) | 0.534 | 1.05(0.82-1.35) | 0.682 | 1.09(0.84-1.42) | 0.499 |
| **Cirrhosis** |  | 0.582 |  | 0.615 |  | 0.794 |
| Q1(0.00-27.15) | 1.00(reference) |  | 1.00(reference) |  | 1.00(reference) |  |
| Q2(27.16-56.70) | 0.98(0.64-1.50) | 0.932 | 0.97(0.64-1.49) | 0.902 | 0.98(0.64-1.52) | 0.937 |
| Q3(56.71-104.55) | 0.79(0.50-1.22) | 0.284 | 0.77(0.49-1.20) | 0.247 | 0.81(0.51-1.28) | 0.358 |
| Q4(104.56-741.65) | 0.80(0.52-1.24) | 0.326 | 0.84(0.54-1.31) | 0.434 | 0.94(0.60-1.49) | 0.804 |

NAFLD: non-alcoholic fatty liver disease, NHANES: National Health and Nutrition Examination Survey, AOR: adjusted odds ratio, Q: quartile.

^a^ Adjusted covariates: A model: age, gender, race, education levels, marital status and household poverty income ratio (PIR); B model: A model plus body mass index (BMI), smoking status, vigorous recreational activities; C model: B model plus hypertension, diabetes, kidney failure, hyperlipemia, hyperuricemia and metabolic syndrome.
