# Supplementary material for: Iron metabolism mediates the relationship between Vitamin C and hepatic steatosis and fibrosis in NAFLD
Source: Front Nutr. 2022 Sep 8;9:952056. doi: 10.3389/fnut.2022.952056 (PMC9494736; doi:10.3389/fnut.2022.952056)
Supplement: Supplementary file 4 [file Data_Sheet_1.docx]

**Supplementary Methods**

**LUTE**

During the 2017-2018 NHANES cycle, liver transient elastography was performed to assess the degree of liver steatosis and sclerosis. All NHANES health technicians and examiners completed a 2-day training program using FibroScan® and passed the exam. MEC staff are regularly supervised by investigators and medical epidemiologists specializing in chronic liver disease, thus ensuring the quality of LUTE.

Based on a recent study, Eddowes et al, We considered median CAP values ≥ 274 dB/m, 290 dB/m, and 302 dB/m to be indicative of S1, S2, and S3 steatosis, respectively[^1^](#_ENREF_16). Combining the results of the MRI-PDFF analysis, we used 288 db/m as the threshold for diagnosing NAFLD[^2^](#_ENREF_17).Median stiffness values ≥8.2 kPa, ≥9.7 kPa, and 13.6 kPa were considered as markers of significant fibrosis (≥F2), advanced fibrosis (F3), and cirrhosis (F4), respectively[^1^](#_ENREF_16).

**Other covariates**

To create a realistic fit model as possible, potential confounders were also included as covariates in our study, which include age, sex (male or female), race (non-Hispanic white, non-Hispanic black, Mexican American, Hispanic, and other races), education level (below high school, high school or equivalent, college or above), marital status (married and single), and household poverty-to-income ratio (PIR) (≤1. 30, 1.31-3.5, >3.5, and others), BMI (<25 kg/m2 and ≥25 kg/m2), strenuous recreational activity (yes or no), moderate recreational activity (yes or no), smoking status, history of hypertension, diabetes, and chronic kidney disease. Smoke exposure (active or passive smoking) status was determined by serum cotinine concentration with a cut-off value of 14 ng/ml. The diagnoses of hypertension, diabetes mellitus, and chronic renal failure in the study subjects either met the clinical criteria or were based on the medical history (history of relevant pharmaceutical use) from the questionnaire.

**Definition metabolic disorders**

Information regarding diabetes, hypertension, and chronic renal failure was based on self-report questionnaires. Hypertension was defined as systolic blood pressure (SBP) > 140 mmHg or diastolic blood pressure (DBP) > 90 mmHg^3^. Diabetes was diagnosed as fasting plasma glucose (FPG) levels ≥7.0 mmol/l or 2h-hour postload blood glucose(PBG) ≥11.1 mmol/l^4,5^. Dyslipidemia was defined as fasting plasma triglyceride (TG)≥1.7 mmol/l, fasting high-density lipoprotein cholesterol (HDL-C)<1.04 mmol/l, low-density lipoprotein cholesterol (LDL-C)≥3.37 mmol/l or total cholesterol (TCH)≥5.18 mmol/l^6^. Hyperuricemia was defined as serum uric acid (UA) ≥ 7 mg/dl (≥417 µmol/L) in men and ≥ 6 mg/dl (≥357 µmol/L) in women^7^. Additionally, MetS was defined by three out of four components as follows^8^:1) overweight and(or) obesity (body mass index (BMI)≥25 kg/m2), 2) FPG ≥6.1 mmol/l and(or) PBG ≥7.8 mmol/l and(or) diagnosed diabetes, 3) hypertension: BP ≥140/90 mmHg or positive history of hypertension or on any hypertensive treatment, 4) dyslipidemia.

**Supplementary Results**

**Comparison of ferritin and VC levels with or without corresponding metabolic disorders**

Serum ferritin levels were significantly higher in subjects with hypertension, diabetes, dyslipidemia, hyperuricemia, or MetS than in subjects without corresponding metabolic disorders (all P<0.001). VC was significantly lower in subjects with diabetes, dyslipidemia, hyperuricemia, or MetS without corresponding metabolic disorders (all P<0.001).VC was lower in subjects with hypertension than in those without hypertension(P>0.05), as shown in Supplementary Fig. 2.

**Supplementary Reference**

1．Eddowes PJ, Sasso M, Allison M *et al*. Accuracy of FibroScan Controlled Attenuation Parameter and Liver Stiffness Measurement in Assessing Steatosis and Fibrosis in Patients With Nonalcoholic Fatty Liver Disease. Gastroenterology 2019; 156:1717-1730.

2. Caussy C, Alquiraish MH, Nguyen P *et al*. Optimal threshold of controlled attenuation parameter with MRI-PDFF as the gold standard for the detection of hepatic steatosis. Hepatology 2018; 67:1348-1359.

3. Giles TD, Materson BJ, Cohn JN *et al*. Definition and classification of hypertension: an update. J Clin Hypertens (Greenwich) 2009; 11:611-614.

4. American Diabetes A. 2. Classification and Diagnosis of Diabetes: Standards of Medical Care in Diabetes-2020. Diabetes care 2020; 43:S14-S31.

5. Ma B, Chen Y, Sheng C *et al*. Low 25-hydroxyvitamin D is associated with arterial stiffness in Chinese with Type 2 diabetes mellitus. Eur J Clin Nutr 2021.

6. Onat A, Hergenc G, Yuksel H *et al*. Neck circumference as a measure of central obesity: associations with metabolic syndrome and obstructive sleep apnea syndrome beyond waist circumference. Clin Nutr 2009; 28:46-51.

7. Ryu KA, Kang HH, Kim SY *et al*. Comparison of nutrient intake and diet quality between hyperuricemia subjects and controls in Korea. Clin Nutr Res 2014; 3:56-63.

8. Alberti KG, Eckel RH, Grundy SM *et al*. Harmonizing the metabolic syndrome: a joint interim statement of the International Diabetes Federation Task Force on Epidemiology and Prevention; National Heart, Lung, and Blood Institute; American Heart Association; World Heart Federation; International Atherosclerosis Society; and International Association for the Study of Obesity. Circulation 2009; 120:1640-1645.
