# Supplementary material for: Iron metabolism mediates the relationship between Vitamin C and hepatic steatosis and fibrosis in NAFLD
Source: Front Nutr. 2022 Sep 8;9:952056. doi: 10.3389/fnut.2022.952056 (PMC9494736; doi:10.3389/fnut.2022.952056)
Supplement: Supplementary file 5 [file Data_Sheet_2.docx]

**Supplementary Figure**

**Figure S1.** Schematic flow diagram of exclusion criteria for study cohort.

**Figure S2.** Serum ferritin and vitamin C levels between metabolic disorders and non-corresponding metabolic disorders. * *p* < 0.05, ** *p* < 0.01, *** *p* < 0.001
